# Considering the Effects and Maternofoetal Implications of Vascular Disorders and the Umbilical Cord

**DOI:** 10.3390/medicina58121754

**Published:** 2022-11-29

**Authors:** Lara Sánchez-Trujillo, Cielo García-Montero, Oscar Fraile-Martinez, Luis G. Guijarro, Coral Bravo, Juan A. De Leon-Luis, Jose V. Saez, Julia Bujan, Melchor Alvarez-Mon, Natalio García-Honduvilla, Miguel A. Saez, Miguel A. Ortega

**Affiliations:** 1 Department of Medicine and Medical Specialities, Faculty of Medicine and Health Sciences, University of Alcalá, 28801 Alcalá de Henares, Spain; 2 Ramón y Cajal Institute of Sanitary Research (IRYCIS), 28034 Madrid, Spain; 3 Deparment of Pediatrics, Hospital Universitario Principe de Asturias, 28801 Alcalá de Henares, Spain; 4 Department of Systems Biology, Faculty of Medicine and Health Sciences (Networking Research Center on for Liver and Digestive Diseases (CIBEREHD)), University of Alcalá, 28801 Alcalá de Henares, Spain; 5 Department of Public and Maternal and Child Health, School of Medicine, Complutense University of Madrid, 28040 Madrid, Spain; 6 Department of Obstetrics and Gynecology, University Hospital Gregorio Marañón, 28009 Madrid, Spain; 7 Health Research Institute Gregorio Marañón, 28009 Madrid, Spain; 8 Department of Biomedicine and Biotechnology, Faculty of Medicine and Health Sciences, University of Alcalá, 28801 Alcalá de Henares, Spain; 9 Immune System Diseases-Rheumatology and Internal Medicine Service, University Hospital Príncipe de Asturias, CIBEREHD, 28806 Alcalá de Henares, Spain; 10 Pathological Anatomy Service, Central University Hospital of Defence-UAH Madrid, 28801 Alcala de Henares, Spain

**Keywords:** umbilical cord, vascular malperfusion, pre-eclampsia, chronic venous disease

## Abstract

The umbilical cord is a critical anatomical structure connecting the placenta with the foetus, fulfilling multiple functions during pregnancy and hence influencing foetal development, programming and survival. Histologically, the umbilical cord is composed of three blood vessels: two arteries and one vein, integrated in a mucous connective tissue (Wharton’s jelly) upholstered by a layer of amniotic coating. Vascular alterations in the umbilical cord or damage in this tissue because of other vascular disorders during pregnancy are worryingly related with detrimental maternofoetal consequences. In the present work, we will describe the main vascular alterations presented in the umbilical cord, both in the arteries (Single umbilical artery, hypoplastic umbilical artery or aneurysms in umbilical arteries) and the vein (Vascular thrombosis, aneurysms or varicose veins in the umbilical vein), together with other possible complications (Velamentous insertion, vasa praevia, hypercoiled or hypocoiled cord, angiomyxoma and haematomas). Likewise, the effect of the main obstetric vascular disorders like hypertensive disorders of pregnancy (specially pre-eclampsia) and chronic venous disease on the umbilical cord will also be summarized herein.

## 1. Introduction

The umbilical cord is an anatomical structure composed of two arteries and a vein covered by Wharton’s jelly derived from allantois, which in turn is upholstered by a layer of amniotic coating [1]. The umbilical cord connects the foetus and the placenta and ensures adequate nutrition, foetal oxygenation, and proper waste elimination. The integrity of the maternal-foetal circulation is essential for the correct development and survival of the foetus. If foetal oxygenation is compromised, foetal hypoxia can affect essential systems such as the cardiovascular system or the central nervous system. Abnormalities or complications that affect these functions involve foetal and neonatal compromise and increase perinatal morbidity and mortality [2].

Both umbilical arteries arise from the internal iliac arteries and are responsible for returning deoxygenated blood from the foetus to the mother. The two arteries converge in the chorionic arteries of the placenta, and their position in the cord is variable. At the histological level, they are characterized by a small lumen comprising a muscular middle layer and an external circular layer and lacking an internal elastic lamina. There is a variant called the single umbilical artery in which there is only one umbilical artery, which can be the result of aneuploidies or congenital anomalies or simply an incidental finding.

The umbilical vein results from the convergence of the chorionic veins and is responsible for the supply of oxygenated blood to the foetus. It is characterized by a wider lumen, with an internal elastic limiting layer and a lax muscular layer in a circular arrangement. During embryogenesis, a right umbilical vein develops that normally degenerates during embryonic development but can persist as a variant in the form of a supernumerary vessel. The umbilical vein connects with the systemic circulation of the foetus through the ductus venosus, which drains into the inferior vena cava. When the cord is detached after birth, the structures contained in the cord sheath remain at the base. The closed blood vessels remain permeable during the first weeks of life. Finally, the umbilical arteries will be defined at the lateral umbilical ligaments, the umbilical vein at the round ligament, and the ductus venosus at the ligamentum venosus.

Wharton’s jelly is derived from mesoblastic cells of the embryonic pedicle and is composed of a hydrophilic extracellular matrix that is rich in water, proteoglycans, and hyaluronic acid. Wharton’s jelly provides supportive and protective functions against compression.

The umbilical cord usually inserts in the placenta centrally or eccentrically, which is considered a normal cord insertion. However, there are insertion abnormalities such as marginal insertion, velamentous insertion, or vasa praevia [1].

Velamentous insertion of the umbilical cord consists of the divergence of umbilical vessels, unsupported by the umbilical cord or placental tissue, as they traverse amnion and chorion before reaching the placenta [3]. It is characterized by the presence of membranous umbilical vessels in the region of placental insertion, little Wharton jelly and susceptibility to compression with the danger of hemorrhage and fetal exsanguination. Vasa praevia consists of an anomaly of the umbilical vessels that cross the membranes of the low uterine segment, unsupported by umbilical cord or placental tissue, with a high risk of rupture of the vessels [3].

Ultrasound examination of the umbilical cord can be performed from the eighth gestational week and is key during prenatal follow-up [4]. There is no consensus about umbilical cord examination among the different societies’ guidelines. The International Society of Ultrasound in Obstetrics and Gynecology, do not recommend checking specifically for possible umbilical cord abnormalities [5]. However, the American Institute of Ultrasound in Medicine (AIUM) guidelines highlight the importance of umbilical cord ultrasound examination between second and third-ultrasound examinations [4].At the anatomic level, its foetal and placental insertion, number of vessels, length, diameter, coiling, and vascular anomalies are important [4].

The average thickness of the cord varies and depends on the length of gestation. A cord with a diameter of less than 1 cm is considered thin [6].

The length of the cord is variable between sexes and gestational age; in term gestations, cords shorter than 35 cm are considered short, and those longer than 70 cm are considered long [6].

Coiling corresponds to the winding pattern of the umbilical arteries around the umbilical vein; 1–3 coils per 10 cm of length is considered normal [1]. In most cases, the pattern is to the left and is evaluated by calculating the coiling index (inverse of the distance separating two spiral turns).

Correct foetal growth and development are also determined by correct placental development. Dysregulation of cell differentiation during placental angiogenesis implies an alteration in the primitive foetal circulation, which may indicate abnormal intrauterine growth [7]. The perinatal and neonatal implications of incorrect placental development vary greatly depending on its severity.

Several factors have been linked to changes in foetal blood flow, including the presence of vascular alterations during pregnancy, which in turn encompass anomalies and vascular alterations in the umbilical cord [8].

## 2. Vascular Alterations of the Umbilical Cord and Its Impact on the Foetus and Newborn

### 2.1. Arterial Vascular Alterations of the Umbilical Cord

Single umbilical artery (SUA) is a variation of cord anatomy in which only a single umbilical artery is present. The absence of the left umbilical artery is more frequent than the absence of the right artery. SUA occurs in approximately 0.5–5% of spontaneous pregnancies, although it depends on the population studied [9]. It is usually the result of an atresia or secondary atrophy of one of the arteries, but it may also be due to a primary agenesis of an umbilical artery or the persistence of the single allantoic artery that originates the umbilical arteries. It can be properly diagnosed with a color Doppler ultrasound of the paravesical umbilical vessels [3].

There is no clear relationship between SUA and certain foetal or neonatal pathologies, although studies suggest increased risks of preterm delivery, caesarean section, low birth weight, small newborn for gestational age and admission to the NICU [10]. The association of SUA with other chromosomal or anatomical abnormalities may also imply changes in foetal and neonatal development [9,11]. The highest incidence of malformation associated has been found in the urinary sytem, cardiovascular system and digestive system [9] If these malformations are present, a genetic testing should be performed [9] such as amniocentesis for karyotype [11].

A similar anomaly is hypoplastic umbilical artery, in which two umbilical arteries are present but one has a significantly smaller diameter than the other, with an artery-to-artery diameter difference of more than 50 per cent [12], which increases blood flow resistance. It can be explained by an atrophy of an artery in late pregnancy. Its association with other abnormalities also affects foetal and neonatal prognosis [12]. Some abnormalities found included trisomy 18, polyhydramnios, congenital heart disease, and fetal growth restriction [12].

Supernumerary vessels are rare in humans, and it is usually a result of the persistence of the right umbilical vein.

Aneurysms in umbilical arteries have also been described. They are a very rare condition and are identifiable by the turbulent pulsatile flow at the ultrasound level. They usually occur together with SUA [10] and are detected in areas near the placental insertion site that are less protected by Wharton’s jelly, usually during the second or third trimester of gestation. They are associated with delayed intrauterine growth, SUA, aneuploidy like trisomy 18, cardiac abnormalities and foetal demise [13]. When aneurysm is detected a detailed ultrasaound examination with fetal echocardiography and karyotype should be considered, as well as early delivery [13].

### 2.2. Venous Vascular Disorders of the Umbilical Cord and Their Impact on the Foetus and Newborn

Vascular thromboses (umbilical cord thrombi) mainly affect the umbilical vein and have been related to other cord abnormalities such as anomalous venous insertion of the cord, an excess of cord coiling, long cords, narrowed cord and little Wharton jelly [14]. They are related to FGR (Fetal Growth Restriction), foetal demise and hypoxic-ischemic encephalopathy [14], so fetus should be closely monitored and a cesarean section surgery should be recommended even without delay [14,15].

These thromboses can be favoured by aneurysms or varicose veins in the umbilical vein, which are identifiable at the ultrasound level as turbulent nonpulsatile flows in areas of dilation. They are more frequent than umbilical artery aneurysms. Vascular thromboses are diagnosed by visualizing dilations greater than 9 mm in diameter or with a diameter greater than 50% of the unaffected vessel and can be intra- or extra-abdominal [16]. Maternal coagulation disorders, vascular endothelial damage and elevated blood glucose have been proposed as possible determining factors to the formation of thrombosis [14] however, the pathogenesis has not been fully elucidated.

Umbilical vain varix is a focal dilatation of the intrabdominalumbilical vein, which has a varix diameter at least 50% wider than the diameter of the intrahepatic umbilical vein [17]. It appears as a fusiform cystic structure.The presence of umbilical venous varices as the only alteration does not usually have foetal repercussions [18]. However, in some studies, the presence of intra-amniotic varicose veins is also related to an increased risk of intra-amniotic haemorrhage, low birth weight and foetal demise [17,19] so fetal monitoring is highly recommended.

### 2.3. Other Vascular Disorders

The insertion of the umbilical cord is almost always central or paracentral and coincides with the anchorage of the amnion. Velamentous insertion of the umbilical cord consists of the divergence of umbilical vessels, unsupported by the umbilical cord or placental tissue, as they traverse amnion and chorion before reaching the placenta [3]. It is characterized by the presence of membranous umbilical vessels in the region of placental insertion, little Wharton jelly and susceptibility to compression. Vasa praevia consists in an anomaly of the umbilical vessels that cross the membranes of the low uterine segment, unsupported by umbilical cord or placental tissue, with a hight risk of rupture of the vessels [3]. It occurs in 1% of pregnancies [6] and it is more frequent in twin pregnancies [20]. Membrane rupture can cause vessel rupture with a risk of exsanguination and foetal demise. Flow compression can translate into placental infarcts and limb amputations [21,22,23,24,25]. In addition, the risks of low birth weight and perinatal death are increased [20,26].

Although prenatal diagnosis is difficult, the coiling pattern of the umbilical vessels and its relationships with venous percussion and fetoplacental blood flow have also been studied. A hypercoiled or hypocoiled cord has been associated with increased risks of adverse perinatal events and foetal demise [27].

A hypocoiled or hypercoiled cord has also been associated with increased risks of preterm childbirth, loss of foetal well-being, meconium in amniotic fluid, Apgar > 7, small for gestational age, foetal and cardiac abnormalities, foetal demise and NICU admission [28]. The coil pattern of the umbilical cord also seems to have implications for fetoplacental flow, as cords with segmented patterns and linked patterns may result in chronic foetal vascular obstruction and stillbirth [29].

In addition, the absence of proper cushioning by Wharton jelly in thin cords seems to favour vascular compression, with consequent repercussions for foetal flow and uterine growth [30]. A thin umbilical cord with little Wharton jelly has been associated with small placental size and low birth weight; that is, a thin umbilical cord seems to be related to placental insufficiency, intrauterine growth restriction and low birth weight [31,32,33,34].

Regarding the length of the umbilical cord, a short umbilical cord has also been related to a higher incidence of adverse events such as urgent caesarean section or low birth weight [32,34]. A longer cord allows wide foetal movements that can increase the risk of crossed and circular entanglement and true cord knots, which can lead to foetal demise [35].

Angiomyxoma, previously also called haemangioma, is an infrequent tumour that arises from the proliferation of mesenchymal angiogenic cells in close relationship with the umbilical vessels [36]. They are usually incidental ultrasound findings, although they can contribute to the involvement of adjacent vessels, favouring hydrops or cord torsion. They are visualized with solid-cystic, echogenic and vascularized mass lesions, usually located in the area of foetal insertion [37]. In some cases, they have been related to foetal demise due to the risk of compression of vessels, rupture and formation of haematomas that compromise the umbilical flow with the foetus [38].

Haematomas of the cord produced by the extravasation of blood from the umbilical vein to Wharton’s jelly have also been described. Although they are infrequent, they can be spontaneous [39] and have a benign course. However, they are usually associated with invasive procedures, infections or morphological abnormalities [40]. They usually have an isoechoic and heterogeneous appearance on ultrasound. This bleeding can be a cause of loss of foetal well-being, intrapartum asphyxia and hypoxic-ischaemic encephalopathy in the newborn [40]. Some studies relate it to oligoamnios in the third trimester, which can increase susceptibility to cord compression [41]. It has also been related to the performance of amniocentesis in the second trimester and an increased risk of prenatal and perinatal death [41].

### 2.4. Foetal Programming: How Vascular Alterations in the Umbilical Cord Can Impact on the Foetus and Newborn

Vascular alterations of the umbilical cord, among other placental or maternal vascular pathologies such as chorioamnionitis, hypertension or preeclampsia [42,43,44] can affect foetal oxygenation during pregnancy. Foetal hypoxia results in anaerobic metabolism in which organic acids such as lactate and ketoacids are produced, leading to metabolic or mixed acidosis.

Different environmental or non-environmental stimuli that make up the intrauterine environment can affect gene expression in the umbilical cord and placenta [45]. The epigenetic changes produced by DNA methylation in different tissues can be decisive in the development of the umbilical cord, placenta, and therefore in the fetus and newborn [45]. These changes conform the concept of fetal health programming. During pregnancy, the hypoxia produced by these vascular alterations leads to a state of fetal programming that can affect the health of the newborn and subsequent development during childhood and adulthood [46,47], affecting cardiac, cerebral or renal function [46]. This concept of fetal programming is evolving as the mechanisms that explain it become clearer [46].

Foetal vascular malperfusion is one of the main patterns of placental damage and is the second most frequent cause of cerebral palsy. Involvement of the umbilical cord has been associated with greater foetal vascular malperfusion at the distal villous level [8].

The pH of arterial and venous blood extracted from the cord at the time of birth can be useful to identify newborns at higher risk of an adverse event in the first hours of life [48], although the criteria for performing this measurement are not clearly established. A pH lower than 7 is a criterion of neonatal asphyxia [48], although the extraction of the umbilical vein or artery should be taken into account. Although this is closely related to neonatal morbidity and mortality, the consequences for the foetus and newborn vary [49], and most newborns do not present long-term neurological or behavioural alterations [50,51,52].

In addition, elevated lactate is a predictor of short-term neonatal morbidity [53] and is associated with increased risks of moderate-severe encephalopathy, cerebral palsy and other cognitive and neurodevelopment alterations [54].

The Apgar Score is used as a quick assessment of the newborn [55] consisting in the assessment of: heart rate, respiratory effort, muscle tone, color and reflex irritability.

Perinatal risk factors can affect the immediate general condition of the newborn [56]. A reduced value in Apgar score could be a predictor of neonatal mortality, especially in very preterm infants [57,58]. However, it is not appropriate to use it alone to identify asphyxia [55]. Also, a high Apgar score could not be sufficient to identify well being newborns as mild metabolic acidosis could be missed [59].

Some studies show a significant and positive correlation between Apgar score and cord pH values [60,61,62]. This correlation has been proved specially in high-risk pregnancies, where the use of cord pH and Apgar Score could be crucial [56].

## 3. Umbilical Cord Alterations Related to Non-Hypertensive Maternal Diseases

Many pregnant women suffer endocrine disorders before and during pregnancy. These conditions have been identified as major contributors to stillbirth [63].

Diabetes Mellitus and carbohydrate intolerance are some frequent metabolic diseases during pregnancy that could affect the structure of the umbilical cord. Some studies suggest that even with optimum glycemic control, diabetes mellitus may be a cause of placental alterations and vascular dysfunction [64,65,66]. Mothers with gestational diabetes mellitus show a down-regulation of vascular endothelial growth factor A (VEGFA), which has a critical role in angiogenesis, producing an abnormal coiling pattern of the umbilical cord [67]. Histopathologic changes have also been described such as a discontinuous endothelial cell of the intima, extravasation of arterial blood to Wharton’s jelly, thinner vein wall, and larger lumen [68]. Also, hypo-coiling has been described as one of the main abnormal patterns of coiling in gestational diabetes [69].

Nowadays, obesity has become a frequent condition among pregnant women. Usually is accompanied by other important conditions such as hypertension and diabetes. It is one of the most important preventable causes of stillbirth [70]. A recent study suggests that umbilical cord abnormalities may account for approximately one-fourth of the effect of obesity on the risk of stillbirth at term [71]. Umbilical hyper coiling, velamentous and marginal cord insertion, thrombosis, and long cord have been described in obese women and all these complications are common causes of stillbirth [71]. Moreover, low umbilical cord blood pH has been found in obese pregnant women, proving that obesity can be an independent risk factor for fetal acidosis at birth increasing newborn morbimortality [72].

## 4. Hypertensive Disorders and Chronic Venous Disease during Pregnancy: Placental and Umbilical Cord Alterations

### 4.1. Hypertensive Disorders during Pregnancy

Both the placenta and the umbilical cord are vascular structures that can be altered by systemic or local vascular changes, including those produced by hypertensive disorders of pregnancy such as chronic hypertension, pregnancy-induced hypertension, preeclampsia, HELLP syndrome and eclampsia [73].

Pregnancy-induced hypertension has been linked to histopathological changes in umbilical vessels. Specifically, a decrease in the lumen of the umbilical vein has been described, along with thickening of the tunica media, increased elastic fibres and decreased collagen fibres [44]. The haemodynamic alterations resulting from these changes would impact foetal blood flow and the foetus. These vascular histopathological changes produce an increase in resistance to the flow of the uterine artery. Recently, it has been proposed that analysis of flow velocity waveforms using machine learning analysis, could be useful to improve the diagnosis of umbilical cord abnormalities [74].

Preeclampsia is a pregnancy condition in wich new-onset hypertension occurs after 20 weeks of gestation and it is related to severe obstetric complications. If affects 2–8% of pregnancies ant it is associated with complications such as FGR and preterm delivery [43].

Decreases in the venous area and wall thickness of the umbilical cord have been observed in pregnant women with preeclampsia and may impact cardiovascular development in the foetus and newborn [43]. However, other studies have reported increased wall and tunica media thickness and an increase in the wall-luminal ratio [53]; therefore, more studies analysing these structural changes are needed. The utility of Doppler ultrasonography in predicting pre-eclampsia has not been extensively studied [75]. However, some studies show that abnormal Doppler ultrasonography has good overall sensitivity in predicting pre-eclampsia [75]. Some studies have also found relationships of preeclampsia with hypercoiling, marginal and paramarginal insertion, and SUA [73].

### 4.2. Chronic Venous Disease during Pregnancy: Placental and Umbilical Cord Alterations and Their Impact on the Foetus and the Newborn

Chronic venous disease (CVD) is a vascular disorder characterized by increased venous hypertension and insufficient venous return from the lower limbs [76]. The haemodynamic changes that occur during pregnancy, such as vasodilation, compression of iliac veins and venous stasis, favour its development [77,78,79,80]. CVD has been associated with several alterations in placental structure and function [80,81,82]. However, the foetal and neonatal repercussions remain unclear and require comprehensive investigation.

At the placental level, CVD has been linked to changes at the level of placental angiogenesis [80], including increases in lymphangiogenesis and angiogenesis. However, the impacts of CVD on placental function, the foetus and the newborn are still unclear.

Elevations of the markers VEGF, TGF beta and PEDF have been observed in the placentas of pregnant women with CVD [81]. These changes suggest that CVD affects the proper development and functioning of the circulatory system, which ensures the correct supply of nutrients and oxygen to the foetus.

CVD has been linked to an increase in the production of reactive oxygen species (ROS) in the venous wall and plasma of affected patients. Elevation of oxidized NADPH (NOXs) has been linked to placental pathology [83] and hypertensive disorders of pregnancy, such as preeclampsia [84]. This oxidative stress has also been detected in the umbilical cord and umbilical foetal blood [85]. At the umbilical level, increases in the gene and protein expression of NOX-1, NOX-2, iNOS, HIF-1alpha and MDA have been observed [86].

Oxidative stress has been linked to ultrasound and cardiotocographic alterations [87,88] such as intrauterine growth retardation, foetal growth restriction, or preterm delivery. According to the foetal programming hypothesis, this oxidative stress is thought to affect the subsequent development of neonatal pathology [87].

In addition, decreases in the expression of cadherin, cadherin 17 and cadherin 6 in the placentas of pregnant women with CVD have been described [89]. Some studies suggest that cadherins are involved in changes in placentation [90,91,92].

Moreover, pregnancy itself is a proinflammatory state [93,94]. The foetus and neonate are also participants in this proinflammatory state [95]. Some studies have shown that gestational CVD favours this proinflammatory state, as indicated by increases in the levels of proinflammatory cytokines (IL-6, IL-12, TNF-α, IL-10, IL-13, IL-2, IL-7, IFN-γ, IL-4, IL-5, IL-21, IL-23, GM-CSF, chemokines (fractalkine), MIP-3α and MIP-1β) in pregnant women with CVD and in the umbilical cord blood of their newborns [76]. At the foetal and neonatal levels, this proinflammatory profile has been related to multiple pathologies, such as preeclampsia, preterm delivery, and the development of bronchial hyperresponsiveness or overweight during the first years of life and therefore forms part of the so-called “foetal programming” [46,47].

## 5. Conclusions

**Figure 1:**
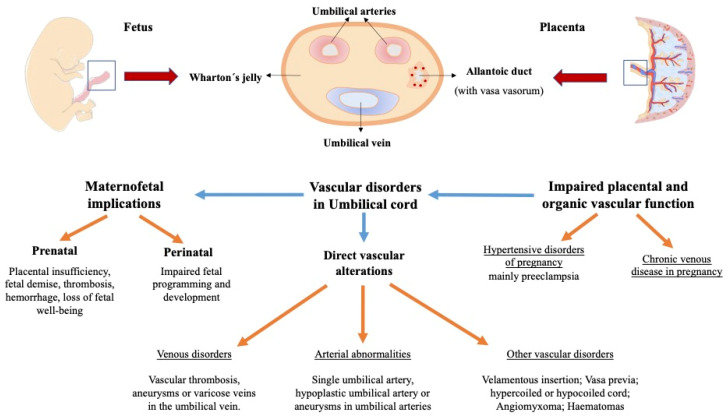
Histological description and vascular alterations observed in the umbilical cord or affecting the umbilical cord, along with the many maternofoetal consequences derived.

The umbilical cord is the link between the foetus and mother and is key in the proper functioning of foetal-placental circulation. As showed in Figure 1, there are plenty possible vascular alterations that may affect the umbilical cord and maternofoetal structures. These vascular alterations of the umbilical cord can compromise or modify foetal blood flow. Hence, changes in the umbilical cord can have a variety of perinatal and neonatal level implications depending on clinical severity as showed in Table 1. Alterations at the level of the umbilical cord are closely related to foetal programming and thus impact the health of the newborn at birth and in later childhood. This array of vascular alterations and CVD emphasizes the need for more studies that allow the establishment of ultrasound, anatomical, histological or plasma markers for the early diagnosis of foetal or prenatal pathologies to prevent foetal and neonatal morbidity and mortality.

## Figures and Tables

**Table 1:** Main vascular alterations of the umbilical cord and their impact on the foetal well-being and the newborn.

Pathology	Cause and Risk Factors	Vascular Alteration	Impact	References
Single umbilical artery (SUA)	Primary agenesis, atresia, or secondary atrophy. Chromosomal abnormalities.	Variation of the anatomy of the cord in which only a single umbilical artery is present. Absence of the left umbilical artery is more frequent. Occurs in 0.5–5% of spontaneous pregnancies.	There is no clear relationship of this isolated variant with a certain foetal or neonatal pathology, although studies suggest that there could be increased risks of preterm delivery, caesarean section, low birth weight, small for gestational age newborn and admission to the NICU. The association of SUA with other chromosomal or anatomical abnormalities (malformation of the urinary system, cardiovascular system and digestive system) may also imply changes in foetal and neonatal development.	[9,10,11]
Umbilical artery aneurysm	SUA. Trisomy 18.	Turbulent pulsatile flow at the ultrasound level. Found in areas close to the placental insertion site that are less protected by Wharton’s jelly, usually during the second or third trimester of gestation.	They are associated with delayed intrauterine growth, aneuploidy and foetal demise.	[13]
Pregnancy-induced hypertension	Risk factors: primary hypertension, renal disease, diabetes, multiple gestations.	Decrease in the lumen of the umbilical vein, thickening of the tunica media, increase in elastic fibres and a decrease in collagen fibres.	Influences foetal blood flow and potentially the foetus	[44,74]
Preeclampsia	Unknown cause.	Decreased venous area and wall thickness in the umbilical cords. Other studies show an increased wall thickness, with increases in the thickness of the tunica media and wall-luminal ratio. Some studies also show relationships of preeclampsia with hypercoiling, marginal and paramarginal insertion and SUA.	Associated with FGR (foetal growth restriction) and preterm delivery. Some studies suggest relationship with hyoercoiling, marginal and paramarginal insertion and SUA.	[43,73,75]
Vascular thrombosis: umbilical cord thrombi.	Maternal coagulation disorders, vascular endothelial damage, elevated blood glucose. Risk factors: hypercoiling, long cord, narrowed cord.	They mainly affect the umbilical vein and are related to vellum insertion of the cord and an excess of cord coiling, with long cords and little Wharton jelly.	They are related to FGR and foetal demise.	[14,15]
Varicose veins or umbilical vein aneurysms	No specific causes and risk factors known.	They are more frequent than umbilical artery aneurysms. Turbulent nonpulsatile flows occur in dilation zones. They are diagnosed by visualizing dilations greater than 9 mm in diameter or with a diameter greater than 50% of the unaffected vessel. They can be intra- or extra-abdominal.	They do not usually have foetal repercussions. Some studies have found an increased risk of intra-amniotic haemorrhage, low birth weight or foetal demise.	[17,18,19]
Velamentous cord insertion and vasa praevia.	No specific causes and risk factors known.	Velamentous insertion of the umbilical cord consists of the divergence of umbilical vessels, unsupported by the umbilical cord or placental tissue, as they traverse amnion and chorion before reaching the placenta with little Wharton jelly and susceptibility to compression. Vasa praevia consists in an anomaly of the umbilical vessels that cross the membranes of the low uterine segment, unsupported by umbilical cord or placental tissue, with a hight risk of rupture of the vessels	The rupture of membranes can cause the rupture of vessels with risk of exsanguination and foetal demise. Flow compression can translate into placental infarcts and limb amputations. In addition, there are increased risks of low birth weight and perinatal death.	[20,21,22,23,24,25,26]
Hypercoiled umbilical cord	No specific causes and risk factors known.	Modifies fetoplacental flow.	Increased risk of adverse perinatal events and foetal demise, increased risk of preterm delivery, loss of foetal well-being, meconium amniotic fluid, Apgar > 7, small for gestational age, foetal and cardiac abnormalities, foetal demise and admission to the NICU.	[28,29]
Hypocoiled umbilical cord	No specific causes and risk factors known.	Modifies fetoplacental flow.	Increased risk of adverse perinatal events and foetal demise, chronic foetal vascular obstruction, stillbirth, increased risk of preterm delivery, loss of foetal well-being, meconium amniotic fluid, Apgar > 7, small for gestational age, foetal and cardiac abnormalities, foetal demise and admission to the NICU.	[28,29]
Thin umbilical cord	No specific causes and risk factors known.	Favours vascular compression with repercussions for foetal flow and uterine growth.	Small placental size, low birth weight, placental insufficiency, intrauterine growth restriction and low birth weight.	[30,31,33,34]
Long umbilical cord	No specific causes and risk factors known.	Greater than 70 cm.	They allow wide foetal movements with greater risk of crossed and circular entanglement and true cord knots, which increases the risk of foetal demise.	[35]
Short umbilical cord	No specific causes and risk factors known.	Less than 35 cm.	Higher incidence of adverse events such as urgent caesarean section or low birth weight.	[32,34]
Umbilical angiomyxoma or haemangioma	Mostly incidental. Risk factors: Hydrops, cord torsion, foetal demise, rupture, haematomas.	Infrequent tumour that arises from the proliferation of mesenchymal angiogenic cells in a close relationship with the umbilical vessels. Solid cystic mass, echogenic and vascularized lesions, usually located in the area of foetal insertion.	Foetal demise due to the risk of compression of vessels, their rupture and formation of haematomas that compromise the umbilical flow to the foetus.	[36,37,38]
Umbilical haematoma	Mostly spontaneous. Risk factors: Invasive procedures (amniocentesis), infections, oligoamnios and morphological abnormalities.	Extravasation of blood from the umbilical vein to Wharton’s jelly.	Loss of foetal well-being, intrapartum asphyxia and hypoxic-ischaemic encephalopathy in the newborn. Oligoamnios in the third trimester. Increased risks of prenatal and perinatal death.	[39,40,41]
Chronic venous disease	Vsodilation, compression of iliac veins and venous stasis during pregnancy, favour its development.		Increases in the gene and protein expression of NOX-1, NOX-2, iNOS, HIF-1alpha and MDA. This oxidative stress has been linked to ultrasound and cardiotocographic alterations [87,88] such as intrauterine growth retardation, foetal growth restriction, or preterm delivery. Some studies have shown that gestational CVD favours this proinflammatory state, as indicated by increases in the levels of proinflammatory cytokines (IL-6, IL-12, TNF-α, IL-10, IL-13, IL-2, IL-7, IFN-γ, IL-4, IL-5, IL-21, IL-23, GM-CSF, chemokines (fractalkine), MIP-3α and MIP-1β). This proinflammatory profile has been related to multiple pathologies, such as preeclampsia, preterm delivery, and the development of bronchial hyperresponsiveness or overweight during the first years of life and therefore forms part of the so-called “foetal programming	[46,47,76,85,87,88]

## Data Availability

Not applicable.
